# Extracellular vesicles for ischemia/reperfusion injury-induced acute kidney injury: a systematic review and meta-analysis of data from animal models

**DOI:** 10.1186/s13643-022-02003-5

**Published:** 2022-09-08

**Authors:** Xia-Qing Li, Jin-Feng Liu, Han Liu, Yu Meng

**Affiliations:** 1 Department of Nephrology, The First Hospital Affiliated to Jinan University, No. 613 Huangpu West Road, Guangzhou, 510630 China; 2 Central Laboratory, The Fifth Hospital Affiliated to Jinan University, Heyuan, China

**Keywords:** Extracellular vesicles, Stem/progenitor cell, Ischemia/reperfusion injury, Acute kidney injury, Systematic review, Meta-analysis

## Abstract

**Background:**

Acute kidney injury (AKI) induced by ischemia/reperfusion injury significantly contribute to the burden of end-stage renal disease. Extracellular vesicles (EVs), especially for stem/progenitor cell-derived EVs (stem/progenitor cell-EVs), have emerged as a promising therapy for ischemia/reperfusion injury-induced AKI. However, their regulatory effects remain poorly understood, and their therapeutic efficiency in clinical trials is controversial. Here, we performed this systematic review and meta-analysis to assess the stem/progenitor cell-EV efficacy in treating ischemia/reperfusion injury-induced AKI in preclinical rodent models.

**Methods:**

A literature search was performed in PubMed, Embase, Scopus, and Web of Science to identify controlled studies about the therapeutic efficiency of stem/progenitor cell-EVs on ischemia/reperfusion injury-induced AKI rodent models. The level of SCr, an indicator of renal function, was regarded as the primary outcome. Meta-regression analysis was used to reveal the influential factors of EV therapy. Sensitivity analysis, cumulative meta-analysis, and assessment of publication bias were also performed in our systematic review and meta-analysis. A standardized mean difference (SMD) was used as the common effect size between stem/progenitor cell-EV-treated and control groups, with values of 0.2, 0.5, 0.8, and 1.0 defined as small, medium, large, and very large effect sizes, respectively.

**Results:**

A total of 30 studies with 985 ischemia/reperfusion injury-induced AKI rodent models were included. The pooled results showed that EV injection could lead to a remarkable sCr reduction compared with the control group (SMD, − 3.47; 95%CI, − 4.15 to − 2.80; *P* < 0.001). Meanwhile, the EV treatment group had lower levels of BUN (SMD, − 3.60; 95%CI, − 4.25 to − 2.94; *P* < 0.001), indexes for tubular and endothelial injury, renal fibrosis (fibrosis score and α-SMA), renal inflammation (TNF-α, IL-1β, iNOS, and CD68 + macrophages), but higher levels of indexes for tubular proliferation, angiogenesis-related VEGF, and reactive oxygen species. However, our meta-regression analysis did not identify significant associations between sCr level and cell origins of EVs, injection doses, delivery routes, and therapy and outcome measurement time (all *P* values > 0.05). Significant publication bias was observed (Egger’s test, *P* < 0.001).

**Conclusion:**

Stem/progenitor cell-EVs are effective in improving renal function in rodent ischemia/reperfusion injury-induced AKI model. These vesicles may help (i) reduce cell apoptosis and stimulate cell proliferation, (ii) ameliorate inflammatory injury and renal fibrosis, (iii) promote angiogenesis, and (iv) inhibit oxidative stress. However, the current systematic review and meta-analysis did not identify significant influential factors associated with treatment effects. More preclinical studies and thoughtfully designed animal studies are needed in the future.

**Supplementary Information:**

The online version contains supplementary material available at 10.1186/s13643-022-02003-5.

## Introduction

Renal ischemia/reperfusion injury is the major cause of intrinsic acute kidney injury (AKI) [1]. It inevitably occurs in clinical procedures, including allograft renal transplantation, partial nephrectomy, and clinical conditions including hypovolemic shock, hypotension, dehydration, and acute tubular necrosis [2, 3]. The pathophysiology of renal ischemia/reperfusion injury consists of renal hemodynamic change, renal hypoxia, inflammatory response, and injuries of kidney endothelial and tubular cells [4]. Due to a lack of efficient therapies, ischemia/reperfusion injury-induced AKI has a high mortality rate reaching 50% and significantly contributes to the burden of end-stage renal disease [5, 6]. Despite significant advancements in technology associated with supportive renal replacement therapy, the prognosis and the mortality rate have not been improved [7]. Accordingly, exploring a new and potent therapeutic strategy is imperative.

An increasing number of investigations in the field of regenerative medicine have focused on the use of stem/progenitor cells in promoting the recovery of AKI. Because of their capability to self-renew and differentiate into functional parenchyma, transplantation of stem/progenitor cells is considered a promising therapeutic strategy for AKI [8, 9]. In particular, mesenchymal stromal cells (MSCs) have garnered an increasing interest for their immunomodulatory and anti-inflammatory properties in ischemia/reperfusion injury-induced AKI [10]. In a previous meta-analysis, the MSC therapy appeared to be more effective in improving impaired renal function in renal ischemia/reperfusion injury model, when compared with cisplatin- and glycerol-induced AKI and 5/6 nephrectomy-induced chronic kidney disease (CKD) animal models [11]. However, concerns regarding the safety issues, such as teratomas formation and chromosomal abnormalities, may limit their clinical applicability [12]. Controversies also remain about the immediate and long-term cell retention rate, the differentiation ability, and the survival rate of the transplanted stem/progenitor cells [13]. Moreover, comorbidities may affect the function of MSCs, such as MSC mitochondrial dysfunction caused by obesity in our previous study, which may limit the therapeutic use of autologous MSCs [14].

Recently, based on the paracrine/endocrine mechanisms of stem/progenitor cells, a novel strategy of “cell-free therapy,” including the administration of extracellular vesicles (EVs), has been exploited in preclinical models of AKI [15, 16]. Extracellular vesicles (EVs), mainly including exosomes and microvesicles, are membrane-bound vesicles released by most cells, including stem/progenitor cells. Exosomes (~ 30–150 nm in size) are formed within multivesicular endosomes and released by exocytosis, and Rab27a regulates exosome secretion [17, 18]; Microvesicles (~ 100–1000 nm) are shed directly by outward budding and fission of the plasma membrane [19]. EVs carry proteins, lipids, carbohydrates, and nucleic acids (including DNA, mRNA, and miRNA), functioning as mediators of intercellular communication [19, 20]. Stem/progenitor cell-derived EVs (stem/progenitor cell-EVs) shuttle functional cargoes capable of reducing tissue injury and/or promoting repair and regeneration in target cells. Previous studies showed that EVs released from adipose-derived mesenchymal stem cells can exert renoprotection by attenuating inflammation and fibrosis [21] and can restore renal function by improving revascularization [22]. In addition, a higher safety profile compared with stem/progenitor cells [23], and the ease of penetrating the glomerular filtration barrier to mediate intra-renal signaling have added to the therapeutic appeal of EVs [24]. Therefore, MSC-derived EVs show a promising potential as a novel therapy for ischemia/reperfusion injury-induced AKI.

To date, many studies investigated the efficacy of stem/progenitor cell-EVs in the treatment of ischemia/reperfusion injury-induced AKI in rodent models. Preclinical animal studies are expected to anticipate the feasibility and efficacy to establish a new therapy. However, contradictory findings have been shown that miRNA released from microvesicles may promote cell apoptosis and participate in renal ischemia/reperfusion injury [25]. Moreover, some important questions remain regarding clinical therapy, including cell origins of EVs, injection doses, delivery routes, and therapy and outcome measurement time. Therefore, we conducted a systematic review and meta-analysis to investigate the therapeutic efficacy of MSC-derived EVs on preclinical rodent models of ischemia/reperfusion injury-induced AKI, and the possible influential factors, ultimately aiming to provide the available clues for clinical trials in future.

## Materials and methods

The PRISMA (Preferred Reporting Items for Systematic Reviews and Meta-Analyses Statement) was used as a writing guide to ensure a standard method to transparent and perform this systematic review and meta-analysis [26].

### Search strategy

We searched PubMed/MEDLINE, Scopus, EMBASE, and the Web of Science for the original articles; The last search was updated on February 24, 2022, for the present study. The search was performed using every possible combination of words: (“extracellular vesicle” OR “EV” OR “exosome” OR “microvesicle” OR “microparticle” OR “MV” OR “shedding vesicle”) AND (“stromal cell” OR “stem cell” OR “SC” OR “progenitor cell” OR “PC”) and (“kidney ischemia–reperfusion injury” OR “renal ischemia–reperfusion injury”). We applied no language restrictions. Bibliographies and reference lists were manually searched to identify additional pertinent studies.

### Eligibility criteria

After the removal of duplicates, the articles were screened using the following inclusion/exclusion criteria. Inclusion criteria: (1) original articles; (2) rodent animal experiment that used rat/mice; (3) object of the study: ischemia/reperfusion injury-induced AKI; (3) intervention group injected with stem/progenitor cell-derived EVs; (4) control group received the same amounts of vehicles alone; and (5) outcome: therapeutic efficacy.

Exclusion criteria: (1) large animal/non-rodent experiments, (2) no control group or inappropriate comparisons, (3) no AKI models, (4) combined therapeutic interventions included other agents with uncertain effects, and (5) article type: comments, letter, reviews, editorial, and case report.

### Protocol of study selection

Two independent reviewers (LXQ and LJF) screened and selected the studies in two phases using eligibility criteria: (1) tile and abstract screening and (2) full-text studies assessment. Any disagreement was resolved through discussion with two other independent reviewers (LH and MY) in a meeting to reach a consensus.

### Outcome measurement

Studies reporting the following outcomes: the primary outcome was the change in kidney function, as measured by serum creatinine (sCr) level during follow-up; secondary outcomes included the blood urea nitrogen (BUN) level, assessment of tubular epithelial cell (TEC) injury (tubular injury score, tubular necrosis, cast, TUNEL, Ki67 and PCNA), endothelial injury(vWF, eNOS), renal fibrosis (fibrosis score and α-SMA), renal inflammation (TNF-α mRNA, IL-1β mRNA, iNOS, and CD68 + macrophages), angiogenesis (VEGF), and oxidative stress (reactive oxygen species and mitochondrial fragmentation).

### Data extraction

The following data were extracted from all eligible studies: publication details, study location, animal species and number, EV cell origins and dose, delivery route, therapy time, and estimation time for kidney function and outcomes (Table 1). For data only reported in figures, a digitalized tool Engauge Digitizer version 4.1 software was used to extract the data. If not reported, study authors were contacted via email to obtain primary experimental data. Excel spreadsheets were built to perform the data extraction table by three independent investigators (LXQ, LJF, and LH), and then, the fourth investigator (MY) checked and synthesis the data.

**Table 1:** Characteristics of the 29 studies included in the meta-analysis

**First author, year**	**N**	**Species/sex**	**Cell origin**	**Doses and size of EVs**	**Isolation method**	**Delivery** r**oute**	**Therapy time**	**Estimation time for kidney function**	**Outcome**
Alzahrani et al. [27]	30	NR. rats/female	BMSC	250 μg Exos	DC, 100,000 g	Arterial	< 1 h	3d and 4w	sCr, BUN, IL-1β, IL-10, VEGF
Burger et al. [28]	92	NOD-SCID mice/male	ECFC	15 μg Exos	DC, 100,000 g and 200,000 g	Intravenous	< 1 h	1d	sCr, BUN, tubular injury score, TUNEL
Cantaluppi et al. [29]	54	Wistar rats/male	EPC	30 μg MVs	DC, 100,000 g	Intravenous	< 1 h	2d and 180 d	sCr, BUN, PCNA, BrdU, TUNEL, Cast, tubular necrosi
Cao et al. [30]	15	FVB/N mice/male	hP-MSC	80 μg EVs	DC, 130,000 g	Intravenous	< 1 h	0d、3d、5d、8d、15d	sCr, BUN, cast, tubular necrosis, Kim-1, mitochondrial fragmentation
Choi et al. [31]	96	FVB/N mice/male	KMSC	2 × 10^7^ MVs	Filtra-centrifugation	Intravenous	< 1 h	1 and 3d	sCr, tubular injury score, PCNA, TUNEL
Collino et al. [32]	32	Wistar rats/male	ADMSC	7.5 × 10^8^ EVs	DC, 100,000 g	Subcapsular	< 1 h	3 d	sCr, BUN, capillary-like structures, Kim-1, PCNA, iNOS, tubular injury score
Collino et al. [33]	14	Wistar rats/male	iPSC and ADMSC	1 × 10^9^ EVs	DC, 100,000 g	Subcapsular	< 1 h	3 d	sCr, BUN, TUNEL, PCNA, tubular injury score, CD68 + macrophages, iNOS, mitochondrial fragmentation
Gatti et al. [34]	28	SD rats/male	BMSC	30 μg MVs	DC, 100,000 g	Intravenous	< 1 h	2d and 6 M	sCr, BUN, PCNA, BrdU, TUNEL, Cast, tubular necrosis
Gu et al. [35]	48	SD rats/male	WJMSC	100 μg EVs	DC, 100,000 g	Intravenous	< 1 h	1d	sCr, BUN, mitochondrial fragmentation, TUNEL
Ju et al. [36]	24	SD rats/male	UCMSC	30 μg MVs	DC, 100,000 g	Intravenous	< 1 h	14 d	sCr, BUN, PCNA, TUNEL
Kilpinen et al. [37]	26	SD rats/male	UCMSC	16 μg MVs	DC, 100,000 g	Arterial	< 1 h	1 and 2 d	sCr, BUN
Li et al. [38]	30	SD rats/male	MSC	100 μg Exos	Precipitation reagents	Arterial	< 1 h	1d	sCr, BUN, TUNEL, TNF-α mRNA
Lin et al. [39]	30	SD rats/male	ADMSC	100 μg Exos	SDS-PAGE Precipitation	Intravenous	3 h	3d	sCr, BUN, tubular injury score, TNF-α, IL-1β
Liu et al. [40]	50	C57BL/6 mice/male	MSC	100 μg EVs	DC, 130,000 g	Intravenous	< 1 h	1d, 3d and 7d	sCr, BUN, Ki67, capillaries density
Ranghino et al. [41]	20	SCID mice/male	Gl-MSC	4 × 10^8^ Exos	DC, 100,000 g	Intravenous	< 1 h	2 d	sCr, BUN, cast, tubular necrosis, PCNA
Shen et al. [42]	18	Balb/c mice/NR	BMSC	200 μg Exos	DC, 100 000 to 110 000 g	Intravenous	10 min	1, 3 and 5 d	sCr, BUN
Vinas et al. [43]	18	FVB mice/male	ECFC	20 μg Exos	DC, 100,000 g	Intravenous	< 1 h	1d	sCr, BUN, PTEN, tubular injury score
Vinas et al. [44]	20	FVB mice/male	ECFC	20 μg Exos	DC, 100,000 g	Intravenous	< 1 h	1d	sCr, BUN, tubular injury score
Wang et al. [45]	144	SD rats/male	BMSC	100 μg Exos	Precipitation reagents	Arterial	< 1 h	2d	sCr, BUN, TUNEL, IL-1β, TNF-α
Wang et al. [3]	120	BALB/c mice/male	BMSC	5 × 10^10^ EVs	DC, 120,000 g	Intravenous	1 h before IRI	8, 16, 24, and 48 h	sCr, BUN
Wu et al. [46]	40	SD rats/male	WJMSC	100 μg MVs	DC, 100,000 g	Intravenous	< 1 h	1d, 2d, 1w and 2w	sCr, BUN, vWF, IL-10, TUNEL, Ki67, α-SMA, TGF-β, CD68 + macrophages
Yuan et al. [47]	48	SD rats/male	iPSC	10^12^ Exos	Precipitation reagents	Intravenous	< 1 h	2d	sCr, BUN, tubular injury score
Yu et al. [48]	20	C57BL/6 mice/male	ESC	100 μg EVs	DC, 100,000 g	left renal cortex	< 1 h	1d, 3d, 5d, 1w and 2w	sCr, BUN, Ki67, cast, Kim-1
Zhang et al. [49]	24	SD rats/male	WJMSC	100 μg MVs	DC, 100,000 g	Intravenous	< 1 h	2w	sCr, BUN, Ki67, TUNEL, ROS, mRNA, fibrosis score, α-SMA
Zhang et al. [50]	24	rats/male	WJMSC	100 μg EVs	DC, 100,000 g	Intravenous	< 1 h	1d	sCr, BUN, tubular injury score, TUNEL, ROS
Zhang et al. [51]	24	C57BL/6 mice/male	UCMSC	100 μg EVs	DC, 100,000 g	Intravenous	< 1 h	2w	sCr, BUN, Brdu, PCNA, α-SMA
Zou et al. [52]	54	SD rats/male	WJMSC	100 μg MVs	DC, 100,000 g	Intravenous	< 1 h	1d, 2d and 2w	sCr, BUN, TUNEL, Ki67, vWF, CD68 + macrophages, IL-10, TNF-α
Zou et al. [53]	60	SD rats/male	UCMSC	100 μg EVs	DC, 100,000	Intravenous	< 1 h	1d	sCr, BUN
Zou et al. [54]	72	NR rats/male	UCMSC	100 μg EVs	DC, 100,000	Intravenous	< 1 h	1d	sCr, BUN, tubular injury score, fibrosis score, TUNEL, Ki67, VEGF
Zhu et al. [55]	15	BALB/c mice/male	BMSC	5 × 10^10^ Exos	DC, 100,000	Intravenous	1 h before IRI	1d	sCr, BUN, tubular injury score,

Species: *SD* Sprague–Dawley

Cell source: *WJMSC*, Wharton Jelly mesenchymal stromal cell; *UCMSC*, human umbilical cord mesenchymal stromal cell; *ADMSC*, adipose-derived MSC; *BMSC*, bone marrow mesenchymal stromal cell; *AT*, adipose tissue; *iPSC*, induced pluripotent stem cell; *HLSC*, human liver stem cell; *EPC*, endothelial progenitor cell; *ECFC*, endothelial colony-forming cell; *ESC*, embryonic stem cell; *UVEC*, umbilical vein endothelial cell; *GI-MSC*, MSC within the glomeruli; *KMSC*, kidney-derived mesenchymal stem cell; *RAPC*, renal artery-derived vascular progenitor cell; *NRK52E*, normal rat kidney cell line 52e; *hP-MSC*, human placenta-derived MSC,

*EVs*, extracellular vesicles; *MV*, microvesicles; *Exo*, exosomes

Isolation method: *DC*, differential centrifugation

Therapeutic Time: *min*, minute; *h*, hour; *d*, day; *w*, week; *m*, month

Outcome estimation: *sCr*, serum creatinine; *BUN*, blood urea nitrogen; *Kim-1*, kidney injury molecule-1; *TEC*, tubular epithelial cell; *TUNEL*, transferase-mediated dUTP nick-end labeling; *PCNA*, proliferating cell nuclear antigen; *BrdU*, Bromodeoxyuridine; *vWF*, von Willebrand Factor; *VEGF*, vascular endothelial growth factor; *IL-1β*, interleukin-1β; *TNF-α*, tumor necrosis factor-α; *α-SMA*, alpha-smooth muscle actin; *ROS*, reactive oxygen species;

*NR*, no report

### Quality assessment

Two independent reviewers (LXQ and MY) assess the methodological quality of each eligible study using a 10-item checklist, adapted from the Collaborative Approach to Meta-Analysis and Review of Animal Data from Experimental Studies (CAMARADES) [56]: A, publication in a peer-reviewed journal; B, control of animals’ temperature; C, randomized treatment allocation; D, blind established model; E, blinded assessment of outcome; F, use of anesthetic without significant intrinsic vascular protection activity; G, appropriate animal model (diabetic, advanced age, or hypertensive); H, reporting of a sample size calculation; I, statement of compliance with animal welfare regulations; and J, statement of potential conflicts of interest.

### Statistical analysis

The effect sizes between stem/progenitor cell-EV-treated and control groups were reported as a pooled standardized mean difference (SMD) according to Cohen’s *d* statistic [57], with the 95% confidence interval (CI). SMD values of 0.2, 0.5, 0.8, and 1.0, respectively, correspond to small, medium, large, and very large effect sizes. The SMD was used because the different measures were employed by many included studies to assess the same outcomes. The random-effect analytical model was used for the analyses. Statistical heterogeneity across studies was assessed using the *I*^2^ statistic. *I*^2^ > 50% indicated significant heterogeneity [58]. The sensitivity analysis was conducted to assess the robustness of results. The subgroup analysis based on cell origins of EVs (mesenchymal stromal/stem cell and progenitor cell) was performed to investigate potential sources of between-study heterogeneity. Meta-regression analyses were carried out focused on cell origins of EVs, injection doses, delivery routes, and therapy and outcome measurement time. A cumulative meta-analysis was performed to explore changes in the results over time. To detect the presence and extent of publication bias, we use the funnel plot, Egger tests, and trim and fill. The data were pooled and analysis using RevMan 5.3 and Stata 12.0/SE statistical software.

## Results

### Search results and study selection

**Fig. 1:**
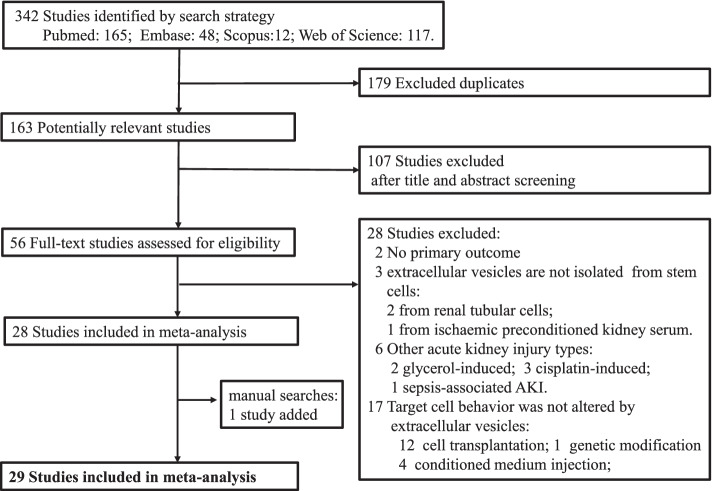
Flow chart of the study selection process

According to the search strategy, a total of 364 studies were identified in the 4 databases. Among them, 58 studies were eligible for full-text review after reviewing the title and abstract. Then, 29 studies were deemed suitable for statistical analysis. One study was added by manually searching the reference lists of eligible studies [59]. Overall, 30 studies were included in our final meta-analysis. The flow chart for this process is shown in Fig. 1.

### Study characteristics

The included studies contained 56 comparisons, and 985 ischemia/reperfusion injury-induced AKI rodent animals (12 studies used mice and 18 used rats). Specifically, 494 animals in the stem/progenitor cell-derived EV group and 491 animals in the control group. EVs were frequently isolated from stem cells (23/30 from mesenchymal stem cells, 2/30 from pluripotent stem cells, and 1/30 from embryonic stem cells) and progenitor cells (4/29). Doses of EVs are varied and can be summarized as high dose (≥ 100 ug or 5 × 10^10^ particles) in 19 studies and low dose MSC (< 100 ug or 5 × 10^10^ particles) in 11 studies. The size of isolated EVs is in the range of 30–1000 nm (mostly 50–350 nm). Surface molecules, such as CD9, CD29, CD44, CD63, CD81, and α4-6 integrins [31, 45], were used to identify and sort out EVs from other microstructures. A variety of microRNAs were reported in EVs, such as miR30 [35], miR126, miR296 [29], and miR486-5p [43]. In most studies, renal ischemia was induced by non-traumatic clamps over the left renal artery for 30 ~ 45 min. Following clamp removal, reperfusion was established. EVs were injected into the animals by intravenous (23/30), arterial (4/30), subcapsule (2/30), or left renal cortex injection (1/30). The EV therapeutic time after renal ischemia/reperfusion injury in most of the studies was < 1 h (27/30). The median time from injection to estimation for kidney function was 2 (range: 0–180) days. Characteristics of the included studies are presented in Table 1.

### Quality assessment

All the studies included in our meta-analysis were peer-reviewed publications. All the renal ischemia/reperfusion injury animals were randomly allocated to the EV treatment group and control group. However, most of the studies did not provide sample size calculation, blinded induction of the ischemia/reperfusion injury model, and blinded assessment of outcome. The results of the quality assessment are shown in Additional file 1.

### Primary outcome (sCr level)

**Fig. 2:**
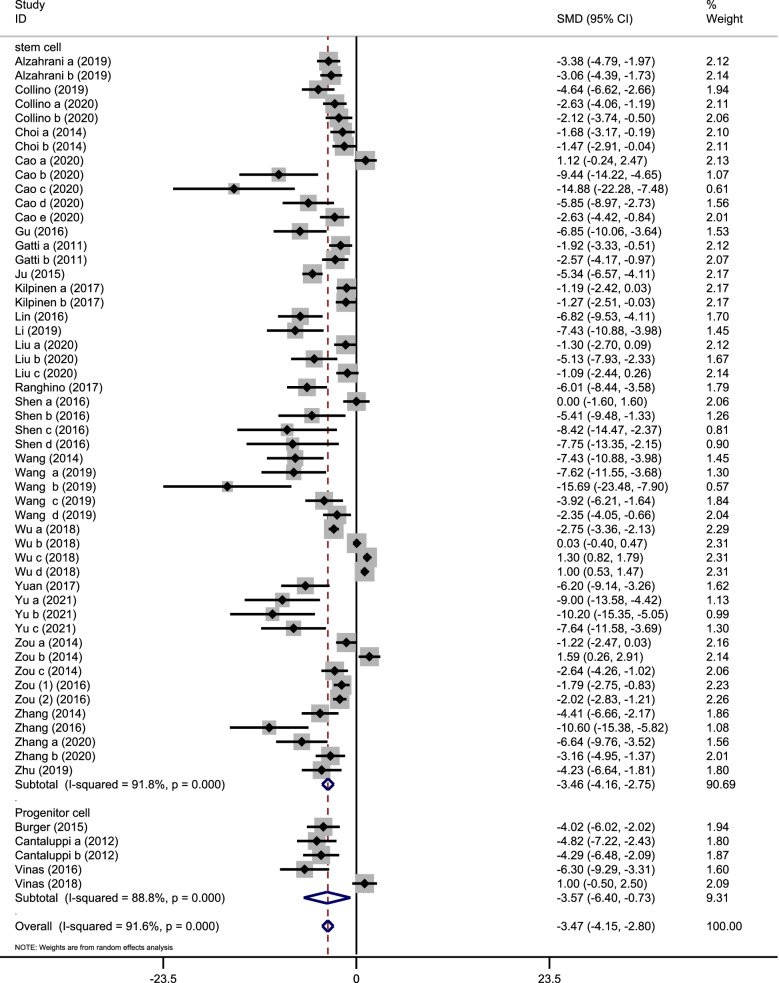
Assessment of Scr levels in a forest plot. The forest plot shows the efficacy in Scr reduction of stem/progenitor cell-derived EVs in the ischemia/reperfusion injury-induced AKI model

All the 30 studies reported the change in sCr level. As is shown in Fig. 2, the pooled result of meta-analysis showed that EV injection could lead to a remarkable sCr reduction, when compared with the control group (SMD, − 3.47; 95%CI, − 4.15 to − 2.80; *P* < 0.001; Fig. 2).

A subgroup analysis was conducted based on the cell origin of EVs and showed that the pooled sCr of each subgroup was consistent with the overall results. Meanwhile, the efficacy in Scr reduction of stem cell-derived EVs (SMD, − 3.46; 95%CI, − 4.16 to − 2.75; *P* < 0.001; Fig. 2) was similar to progenitor cell-derived EVs (SMD, − 3.57; 95%CI, − 6.40 to − 0.73; *P* < 0.001; Fig. 2). A cumulative meta-analysis by publication year was performed and showed that the pooled result did not change over time (see Additional file 3).

### Secondary outcome

#### BUN level

**Fig. 3:**
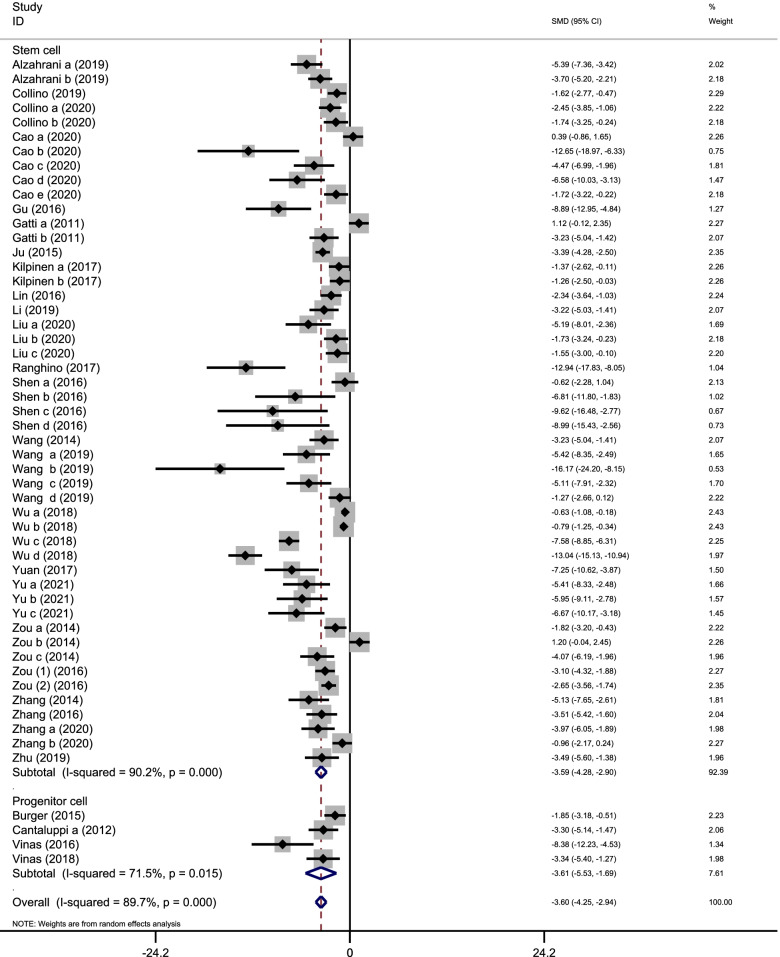
Assessment of BUN levels in a forest plot. The forest plot shows the efficacy in BUN reduction of stem/progenitor cell-derived EVs in the ischemia/reperfusion injury-induced AKI model

Twenty-seven studies were included to compare the level of BUN between the stem cell-derived EV group and control group. The pooled data showed that EVs can significantly reduce the BUN level (SMD, − 3.60; 95%CI, − 4.25 to − 2.94; *P* < 0.001; Fig. 3). The subgroup analysis indicated that EVs isolated from both stem cells (SMD, − 3.59; 95%CI, − 4.28 to − 2.90; *P* < 0.001; Fig. 3) and progenitor cell (SMD, − 3.61; 95%CI, − 5.53 to − 1.69; *P* = 0.015; Fig. 3) are effective in reducing the level of BUN.

### TEC injury assessment

Twenty-five studies were included to assess the effect of stem cell-derived EVs on tubular injury. Among them, tubular injury score was reported in 12 studies, tubular necrosis in 5, cast in 6, TUNEL in 15, Ki67 in 6, PCNA in 8, and BrdU in 3 (see Table 1). Specifically, the EV treatment tubular group has a lower tubular injury score (SMD, − 2.21; 95%CI, − 2.84 to − 1.59; *P* < 0.00001; Table 2) and tubular injury biomarker Kim-1(SMD, − 7.29; 95%CI, − 12.45 to − 2.13; *P* = 0.006; Table 2) compared with the control, as well as lower number of tubular necrosis (SMD, − 6.13; 95%CI, − 9.72 to − 2.53; *P* = 0.0008; Table 2) and casts (SMD, − 4.80; 95%CI, − 7.10 to − 2.50; *P* < 0.00001; Table 2). Moreover, EVs could significantly reduce TEC apoptosis, as determined by fewer TUNEL-positive TECs (SMD, − 3.36; 95%CI, − 4.42 to − 2.29; *P* < 0.00001; Table 2). Interestingly, the number of proliferating TECs in the EV group was significantly higher than that in the control group, as assessed by BrdU staining (SMD, 4.19; 95%CI, 2.00 to 6.37; *P* = 0.0002; Table 2), Ki67 staining (SMD, 1.57; 95%CI, 1.00 to 2.13; *P* < 0.00001; Table 2) and PCNA staining (SMD, 5.60; 95%CI, 3.71 to 7.49; *P* < 0.00001; Table 2).

**Table 2:** Secondary outcomes

Outcomes	Observation	Study number	SMD (95%CI)	*Q* test (*P* value)	Heterogeneity, *I*^2^ (*P* value)
Tubular injury score	Tubular necrosis, loss of brush border, cast formation and tubular dilatation	12	− 2.21 (− 2.84, − 1.59)	0.002	61% (< 0.00001)
Kim-1	Biomarker for proximal tubular injury	3	− 7.29 (− 12.45, − 2.13)	0.02	74% (0.006)
Tubular necrosis	Histological signs of tubular injury	5	− 6.13 (− 9.72, − 2.53)	< 0.0001	85% (0.0008)
Cast	Histological signs of tubular injury	6	− 4.80 (− 7.10, − 2.50)	< 0.0001	80% (< 0.00001)
TUNEL	TEC apoptosis	15	− 3.36 (− 4.42, − 2.29)	< 0.00001	91% (< 0.00001)
Ki67	TEC proliferation	6	1.57 (1.00, 2.13)	0.007	62% (< 0.00001)
PCNA	TEC proliferation	8	5.60 (3.71, 7.49)	< 0.00001	86% (< 0.00001)
BrdU	TEC proliferation	3	4.19 (2.00, 6.37)	0.09	58% (0.0002)
vWF	A marker of endothelial injury	2	− 3.88 (− 4.38, − 3.37)	0.77	0% (< 0.00001)
VEGF	Angiogenesis-related gene	2	3.32 (2.35, 4.28)	0.21	36% (< 0.00001)
Capillary density	Angiogenic capacity	2	1.47 (0.52, 2.42)	0.34	0% (0.002)
CD68^+^macrophages	Inflammatory response	3	− 7.69 (− 11.14, − 4.24)	< 0.00001	96% (< 0.00001)
iNOS	a biomarker for M1-polarized macrophages	2	− 2.05 (− 3.35, − 0.75)	0.3	16% (0.002)
IL-1β mRNA	pro-inflammatory cytokine	2	− 2.28 (− 4.38, − 0.17)	0.0006	87% (0.03)
TNF-α mRNA	pro-inflammatory molecule	2	− 3.47 (− 5.44, − 1.50)	0.19	41% (0.0005)
Fibrosis score	The degree of interstitial fibrosis	2	− 4.39 (− 6.11, − 2.67)	0.58	0% (< 0.00001)
α-SMA	A myofibroblast marker involved in renal fibrosis	2	− 4.14 (− 6.87, − 1.42)	< 0.00001	95% (0.003)
ROS	Oxidative stress	2	− 1.76 (− 2.61, − 0.92)	0.37	1% (< 0.0001)
Mitochondrial fragmentation	Mitochondrial antioxidant defense	3	− 3.04 (− 4.51, − 1.57)	0.13	47% (< 0.0001)

*Kim-1*, kidney injury molecule-1; *TEC*, tubular epithelial cell; *TUNEL*, transferase-mediated dUTP nick-end labeling; *PCNA*, proliferating cell nuclear antigen; *BrdU*, Bromodeoxyuridine; *vWF*, von Willebrand Factor; *VEGF*, vascular endothelial growth factor; *IL-1β*, interleukin-1β; *TNF-α*, tumor necrosis factor-α; *α-SMA*, alpha-smooth muscle actin; *ROS*, reactive oxygen species

### Endothelial injury assessment

Endothelial injury was also assessed, and 2 studies reporting the level of vWF were recruited [46, 52]. The results showed that stem cell-derived EV group had a lower level of serum vWF compared with the control group (SMD, − 3.88; 95%CI, − 4.38 to − 3.37; *P* < 0.00001; Table 2).

### Renal fibrosis assessment

Four studies were identified to detect the efficacy of stem cell-derived EVs in ameliorating renal fibrosis (2 studies for fibrosis score [49, 54] and 2 for α-SMA [46, 51]). The pooled results indicated that the fibrosis score (SMD, − 4.39; 95%CI, − 6.11 to − 2.67; *P* < 0.00001; Table 2) and α-SMA (SMD, − 4.14; 95%CI, − 6.87 to − 1.42; *P* = 0.003; Table 2) were significantly lower in the EV group when compared with the control group.

### Renal inflammation assessment

Seven studies were included for the assessment of the effect of stem cell-derived EVs on renal inflammation (2 studies for TNF-α mRNA [38, 45], 2 for IL-1β mRNA [27, 45], 2 for iNOS mRNA [32, 33], and 3 for CD68 + macrophages [33, 46, 52]). We found that stem cell-derived EVs could reduce inflammatory responses by decreasing the mRNA levels of inflammatory cytokines, such as TNF-α mRNA (SMD, − 3.47; 95%CI, − 5.44 to − 1.50; *P* = 0.0005; Table 2) and IL-1β mRNA (SMD, − 2.28; 95%CI, − 4.38 to − 0.17; *P* = 0.03; Table 2). Meanwhile, EV injection could reduce the inflammatory infiltration of CD68 + macrophages when compared with the control, and the difference in the number of CD68 + macrophages between the EV group and the control group was notable (SMD, − 7.69; 95%CI, − 11.14 to − 4.24; *P* < 0.00001; Table 2). The treatment with EV could lead to a significantly lower expression of iNOS, a biomarker for M1-polarized macrophages (SMD, − 2.05; 95%CI, − 3.35 to − 0.75; *P* = 0.002; Table 2).

### Angiogenesis assessment

The effect of stem cell-derived EVs on angiogenesis was also assessed, and 2 studies were included [27, 54]. EV group showed a significantly higher expression of the angiogenesis-related marker VEGF than the control group (SMD, 3.32; 95%CI, 2.35 to 4.28; *P* < 0.00001; Table 2). The angiogenic capacity of the EV group was further confirmed by the higher capillary density compared with the control (SMD, 1.47; 95%CI, 0.52 to 2.42; *P* = 0.002; Table 2).

### Oxidative stress assessment

The reactive oxygen species (ROS) level was measured in ischemia/reperfusion injury kidney tissues to assess oxidative stress. Only 2 studies reported ROS levels [49, 50], and the result showed that administration of EVs could decrease the ROS level compared with the control group and thus help alleviate the oxidative stress after renal ischemia/reperfusion injury (SMD, − 1.76; 95%CI, − 2.61 to − 0.92; *P* < 0.0001; Table 2). Moreover, three studies were included for assessing the effect of EVs on the mitochondria. The pooled result indicated that EVs could protect renal cells from oxidative insult through alleviating mitochondrial fragmentation (SMD, − 3.04; 95%CI, − 4.51 to − 1.57; *P* < 0.0001; Table 2).

### Meta-regression and sensitivity analysis

Our multivariable meta-regression analysis did not show significant associations between the levels of sCr or BUN and cell origins of EVs (*P* = 0.363), injection doses (*P* = 0.080), delivery routes (*P* = 0.102), and therapy and outcome measurement time (*P* = 0.495 and *P* = 0.625, respectively) (see Additional file 2). The sensitivity analysis for the sCr level showed that no single study qualitatively influenced the pooled SMDs, suggesting that the results of this meta-analysis were robust (see Additional file 4).

### Publication bias

We tested the potential publication bias for the primary outcomes of sCr level. Significant publication bias was observed, and funnel plot asymmetry is quantified with Egger’s test (*P* < 0.001, Additional file 5). However, further Trim-and-fill analysis showed that the overall results were not significantly changed (i.e., no trimming performed, because data was unchanged) (see Additional file 6). Therefore, this publication bias did not impact the meta-analysis outcomes.

## Discussion

To our knowledge, this is the first preclinical systematic review and meta-analysis providing a comprehensive summary of the effect of stem/progenitor cell-EVs on the rodent ischemia/reperfusion injury-induced AKI model. Our findings confirm that the administration of stem/progenitor cell-EVs is effective in improving renal function in rodent ischemia/reperfusion injury-induced AKI model. These vesicles may help (i) reduce cell apoptosis and stimulate cell proliferation, (ii) ameliorate inflammatory injury and renal fibrosis, (iii) promote angiogenesis, and (iv) inhibit oxidative stress. However, our meta-regression analysis did not identify significant associations between the level of sCr and cell origins of EVs, injection doses, delivery routes, and therapy and outcome measurement time. Therefore, our systematic review and meta-analysis offer significant clues that may help human clinical trial development on EVs and establish new therapeutic modality for ischemia/reperfusion injury-induced AKI.

In previous meta-analyses, researchers have shown a more marked therapeutic effect of EVs on renal failure compared with conditioned medium [60]. Of note, the renoprotective function of EVs was further confirmed in animal models of different types of AKI [61]. EVs derived from various cell sources could significantly reduce the Scr level during AKI (SMD, − 3.71; 95%CI, − 4.32 to − 3.10; *P* < 0.001). Meanwhile, no significant difference was found between stem cell-derived EVs and stem cells. Furthermore, MSC therapy could lead to a greater sCr reduction in ischemia/reperfusion injury-induced AKI, when compared with toxic-ischemic AKI and CKD animal models [11]. On the basis of the previous findings, our focus on the effect of stem/progenitor cell-EVs on ischemia/reperfusion injury-induced AKI and further investigate the underlying mechanisms of EV therapeutic effects.

Renal recovery after ischemia/reperfusion injury-induced AKI includes both tubular and endothelial regeneration [62]. In the present systematic review and meta-analysis, the renoprotective effects of stem/progenitor cell-EVs were further supported by significant reductions in indexes of tubular cell injury (tubular necrosis, cast formation, and apoptosis) and endothelial injury (vWF level) and enhanced tubular proliferation. Angiogenesis was considered as a critical step in tissue regeneration. Our results showed that stem/progenitor cell-EVs may have a pro-angiogenesis effect on the post-ischemia/reperfusion injury kidney, by increasing the expression of VEGF. This vascular trophic activity could help sustain capillary density to prevent microvascular rarefaction after renal ischemia/reperfusion injury. Oxidative stress, inflammation, and apoptosis are important in the early-stage pathophysiology of ischemia/reperfusion injury-induced AKI [33, 63]. In our pooled results, we found an anti-oxidative role of stem/progenitor cell-EVs in renal ischemia/reperfusion injury, as indicated by the decreased ROS level, and the decreased mitochondrial fragmentation. Inflammatory responses caused by oxidative stress during renal ischemia/reperfusion injury are highlighted in many studies [32, 50], and the inflammatory suppression effect of stem/progenitor cell-EVs was also confirmed in our meta-analysis. These vesicles could decrease the mRNA levels of pro-inflammatory cytokines (TNF-α and IL-1β) and lessen the M1-type macrophage polarization and infiltration. In addition to downregulation of pro-inflammatory markers, some in vivo studies have also shown an elevated level of anti-inflammatory cytokine IL-10 after stem/progenitor cell-EVs treated [27, 46]. It was reported that suppression of CX3CL1, a chemo-attractant factor, might be a way for microvesicles to reduce the macrophage infiltration in the ischemia/reperfusion injury kidney [52]. Furthermore, renal fibrosis is the final result of severe ischemia/reperfusion injury-induced AKI [64]. The exposure to ischemia/reperfusion injury contributed to the fibrotic lesions in renal interstitial in the late stage[49]. We found that stem/progenitor cell-EVs display an anti-fibrotic property, as evidenced by decreased fibrosis-score and expression of pro-fibrotic factor α-SMA. Therefore, our systematic review and meta-analysis showed that administration of stem/progenitor cell-EVs exerts renoprotection against ischemia/reperfusion injury through ameliorating pathophysiological processes in both acute and chronic stages.

As a cell-free therapy, EV injection offers many advantages, including high stability and permeability, and low immunogenicity and cytotoxicity [65]. Therefore, the administration of EVs may represent a feasible and safe alternative to cell-based therapy. Most studies have attributed these beneficial effects mostly to their RNA cargo, and RNase treating could also abrogate the effects. Several miRNA candidates have been involved in pathophysiological processes of ischemia/reperfusion injury, such as anti-apoptosis (miR-30[35], miR‐199a‐3p [55], miR-21[66]), pro-angiogenesis (miR-126 and miR-296 [29]), and anti-inflammation (miR-21 [67]). Researchers have found that ligand-receptor interactions, such as CXCR4/SDF-1α interaction, mediate targeting of EVs to the postischemic injured kidneys, leading to increased miRNA levels within proximal tubule and endothelial cells [44]. The miRNAs appear to confer better renoprotective effects to EVs than their parental stem/progenitor cells [55]. Meanwhile, the protective effects of stem/progenitor cell-EVs on renal ischemia/reperfusion injury have been associated with the activation of multiple signaling pathways, including Keap1-Nrf2 [30], PTEN/Akt [43], Nrf2/ARE [50], and Erk1/2 pathways [55]. Signaling events can be initiated by endocytosis depending on specific ligand–receptor interactions or by activation of receptors on the plasma membrane of the target cells.

In our study, the sources of heterogeneity could not be identified by meta-regression analysis. The dose and cell origin of EVs were previously reported to be associated with the therapeutic efficacy of EVs, but our meta-regression analysis did not show statistical significance. The reasons for inconsistent results may be ascribed to the fact that in our meta-analysis, and the eligible studies have differences in sizes of EVs and EV isolated methods. As demonstrated by recent studies, the components of different sizes of EVs are highly heterogeneous. A reassessment of exosome components suggested they are not vehicles of active DNA release [68]. It was reported that miRNAs and DNA are more abundant in exosomes and microvesicles, respectively [69, 70]. In our meta-analysis, different isolated methods were used to identify EVs across different studies. Moreover, the follow-up period was different in our included studies, and the long-term period used in certain studies might have introduced the risks of overestimation of EV viability into our meta-analysis. The lack of blinding in animal studies has been associated with inflated efficacy estimates of EV treatment in original studies. Therefore, these factors may have somehow offset the effects of each other. We believe that our results of meta-regression analysis were more likely influenced by the heterogeneities among original studies than by the real therapeutic efficiency of EVs. In the future, more rigorously designed animal studies are needed to better determine EV efficiency in ischemia/reperfusion injury-induced AKI.

To date, extensive clinical trials have been registered to utilize MSCs for the treatment of different human diseases (clinicaltrials.gov). It is worth mentioning that, in patients with chronic kidney diseases and graft-versus-host disease, administration of MSC-derived EVs significantly improves their clinical outcomes without side effects [71]. In particular, the study focusing on CKD showed that EV therapy could improve renal function (improved eGFR, serum creatinine, and BUN levels) and ameliorate renal inflammation (decreased TNF-α level, whereas increased IL-10) [72]. MSC-EV administration has therapeutic efficiency that is analogous to MSC [73] and effectively avoids the disadvantages of MSC-based therapy. Additionally, in previous studies using healthy pig, a very effective model mimicking many characteristics of human physiology [74], researchers found that nucleic acid and proteins enriched in EVs from adipose-derived mesenchymal stem cells potentially participate in tissue repair and regeneration, by modulating genes associated with anti-inflammatory, pro-angiogenic, and anti-apoptosis [59, 74, 75]. However, there remain significant challenges that need to be addressed to realize the clinical translation. Indeed, one common challenge is the optimization of EV isolation, with special regard to purity, efficiency, and production. Meanwhile, more efforts should be made to maintain EV function and sustain release in the long term. Furthermore, it is essential to establish effective tracking tools to further detect the injected EVs.

### Limitations

First, we did not identify any factors that might influence the therapeutic efficacy of EVs on renal ischemia/reperfusion injury, due to the interstudy heterogeneities. Second, studies included in our meta-analysis used mostly small animal models, which might have been less accurate to represent clinical condition than large animals. Third, much work was done on the EV effects on innate immunity and inflammation during renal ischemia/reperfusion injury, and in fact, more in-depth animal studies are needed to focus on adaptive immunity concerning renal ischemia tolerance [46]. Finally, most of the data were extracted from graphics by Engauge Digitizer software, which may also affect the results.

## Conclusion

Stem/progenitor cell-derived EVs are effective in improving kidney function in rodent ischemia/reperfusion injury-induced AKI model. These vesicles may help (i) reduce cell apoptosis and stimulate cell proliferation, (ii) ameliorate inflammatory injury and renal fibrosis, (iii) promote angiogenesis, and (iv) inhibit oxidative stress. However, the present systematic review and meta-analysis did not identify significant associations between therapeutic efficiency and relevant factors including cell origins of EVs, injection doses, delivery routes, and therapy and outcome measurement time. More preclinical studies and thoughtfully designed animal studies are needed to deeply investigate the EV therapy in ischemia/reperfusion injury-induced AKI.

## Supplementary Information

**Additional file 1.** Quality of eligible studies.

**Additional file 2.** Meta-regression analysis.

**Additional file 3.** Cumulative meta-analysis.

**Additional file 4.** Sensitivity analysis.

**Additional file 5.** Eggers test.

**Additional file 6.** Trim and fill analysis.

## Data Availability

The authors confirm that all data underlying the findings are fully available without restriction. All relevant data are provided in the paper and its Additional files.

## Abbreviations

AKI: Acute kidney injury
IRI: Ischemia/reperfusion injury
EVs: Extracellular vesicles
MV: Microvesicles
Exo: Exosomes
SPC: Stem/progenitor cell
ESRD: End-stage renal disease
MSC: Mesenchymal stromal cells
CKD: Chronic kidney disease
sCr: serum creatinine
BUN: Blood urea nitrogen
TEC: Tubular epithelial cell
SMD: Standardized mean difference
Kim-1: Kidney injury molecule-1
TEC: Tubular epithelial cell
TUNEL: Transferase-mediated dUTP nick-end labeling
PCNA: Proliferating cell nuclear antigen
BrdU: Bromodeoxyuridine
vWF: von Willebrand Factor
VEGF: Vascular endothelial growth factor
IL-1β: Interleukin-1β
TNF-α: Tumor necrosis factor-α
α-SMA: Alpha-smooth muscle actin
ROS: Reactive oxygen species
