# Supplementary material for: Extracellular vesicles for ischemia/reperfusion injury-induced acute kidney injury: a systematic review and meta-analysis of data from animal models
Source: Syst Rev. 2022 Sep 8;11:197. doi: 10.1186/s13643-022-02003-5 (PMC9461206; doi:10.1186/s13643-022-02003-5)
Supplement: Supplementary file 1 — Additional file 1. Quality of eligible studies. [file 13643_2022_2003_MOESM1_ESM.pdf]

Additional file 1: Quality of eligible studies.

Study	A	B	C	D	E	F	G	H	I	J	Total
Alzahrani et al., 2019[42]	√	√	√			√	√		√		6
Burger et al., 2015[65]	√	√	√			√	√		√	√	7
Cantaluppi et al., 2012[34]	√	√	√			√	√		√	√	7
Cao et al., 2020[56]	√	√	√			√	√		√		6
Choi et al., 2014[31]	√	√	√			√	√		√		6
Collino et al., 2019[43]	√	√	√			√	√		√	√	7
Collino et al., 2020[44]	√	√	√			√	√		√		6
Gatti et al., 2011[66]	√	√	√			√	√		√		6
Gu et al., 2016[33]	√	√	√			√	√		√	√	7
Ju et al., 2014[67]	√	√	√			√	√		√		6
Kilpinen et al., 2013[68]	√	√	√			√	√		√		6
Li et al., 2019[41]	√	√	√			√	√		√		6
Lin et al., 2016[69]	√	√	√			√	√		√	√	7
Liu et al., 2020[70]	√	√	√			√	√		√		6
Ranghino et al., 2017[71]	√	√	√			√	√		√		6
Shen et al., 2016[72]	√	√	√			√	√		√		6
Vinas et al., 2016[35]	√	√	√			√	√		√	√	7

Vinas et al., 2018[55]	√	√	√			√	√		√	√	7
Wang et al., 2014[32]	√	√	√			√	√		√		6
Wang et al., 2019[3]	√	√	√			√	√		√		6
Wu et al., 2018[36]]	√	√	√			√	√		√		6
Yuan et al., 2017[73]	√	√	√			√	√		√	√	7
Yu et al., 2021[74]	√	√	√			√	√		√		6
Zhang et al., 2014[38]	√	√	√			√	√		√		6
Zhang et al., 2016[45]]	√	√	√			√	√		√		6
Zhang et al.,2020[40]	√	√	√			√	√		√		7
Zou et al., 2014 [37]	√	√	√			√	√		√	√	6
Zou et al., 2016(1)[75]	√	√	√			√	√		√		6
Zou et al., 2016(2) [39]	√	√	√			√	√		√	√	7
Zhu et al., 2019[52]	√	√	√			√	√		√		6

Abbreviation: A, publication in a peer reviewed journal; B, control of animals' temperature; C, randomized treatment allocation; D, blind established model; E, blinded assessment of outcome; F, use of anaesthetic without significant intrinsic vascular protection activity; G, appropriate animal model (diabetic, advanced age, or hypertensive); H, reporting of a sample size calculation; I, statement of compliance with animal welfare regulations; and J, statement of potential conflicts of interest
