# Supplementary material for: Extracellular vesicles for ischemia/reperfusion injury-induced acute kidney injury: a systematic review and meta-analysis of data from animal models
Source: Syst Rev. 2022 Sep 8;11:197. doi: 10.1186/s13643-022-02003-5 (PMC9461206; doi:10.1186/s13643-022-02003-5)
Supplement: Supplementary file 2 — Additional file 2. Meta-regression analysis. [file 13643_2022_2003_MOESM2_ESM.pdf]

Additional file 2. Meta-regression analysis

Variable	Coefficient	SE	95% CI	*p* value
Cell origin	-1.591045	1.733625	-5.073134 to 1.891044	0.363
Doses of EVs	-2.243208	1.254497	-4.762939 to 0.2765232	0.080
Delivery route	-1.992918	1.195225	-4.393598 to 0.4077611	0.102
Therapy time	0. 4904973	0.7136227	-0.9428561 to 1.923851	0.495
Estimation time	0. 0066529	0.0135385	-0.02054 to 0.0338457	0.625

SE, standard error; CI, Conf. Interval
