# Supplementary figures and images for: Extracellular vesicles for ischemia/reperfusion injury-induced acute kidney injury: a systematic review and meta-analysis of data from animal models
Source: Syst Rev. 2022 Sep 8;11:197. doi: 10.1186/s13643-022-02003-5 (PMC9461206; doi:10.1186/s13643-022-02003-5)

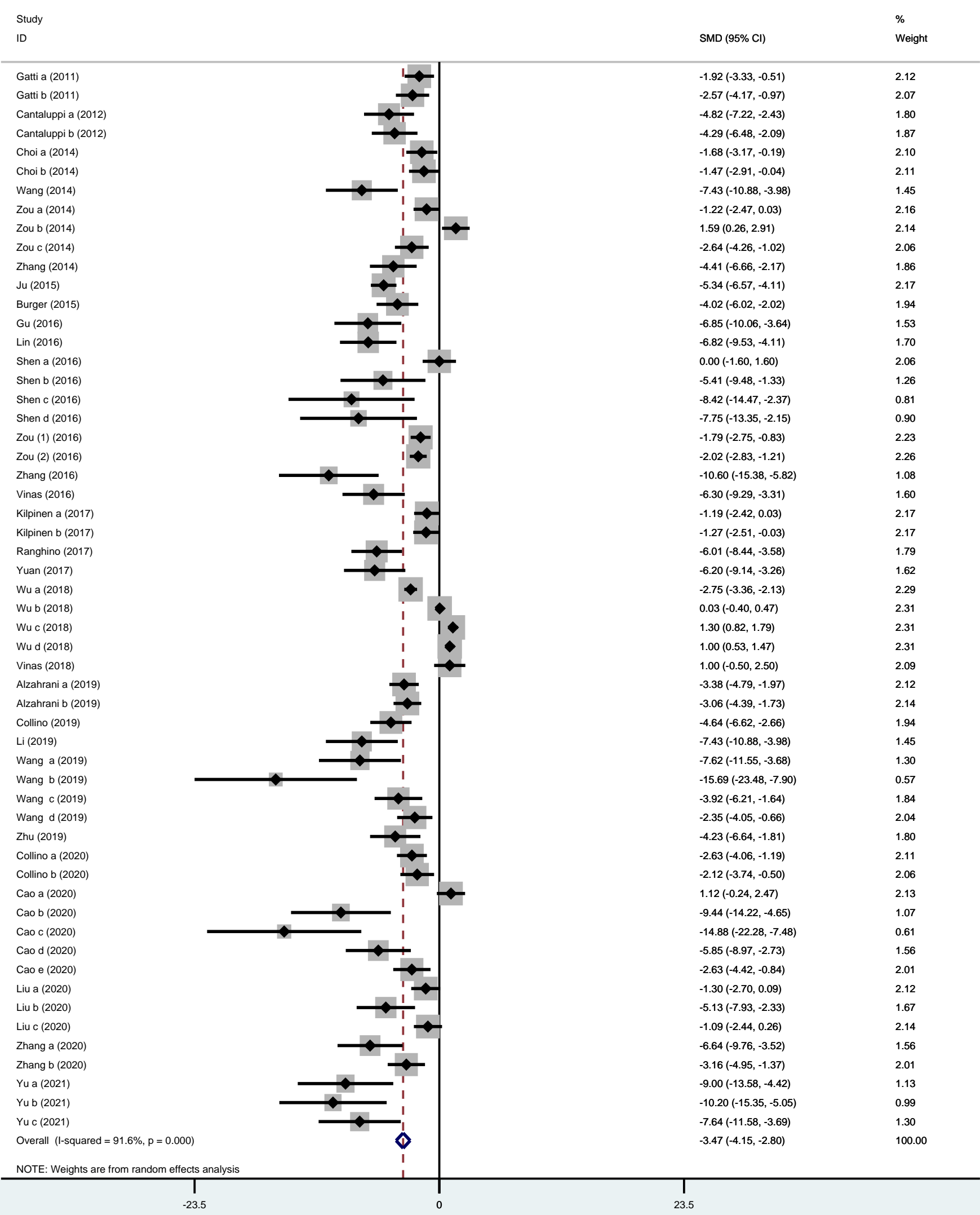

Supplement: Supplementary file 3 — Additional file 3. Cumulative meta-analysis. [file 13643_2022_2003_MOESM3_ESM.pdf]

Meta-analysis estimates, given named study is omitted

| Lower CI Limit      ○ Estimate      | Upper CI Limit

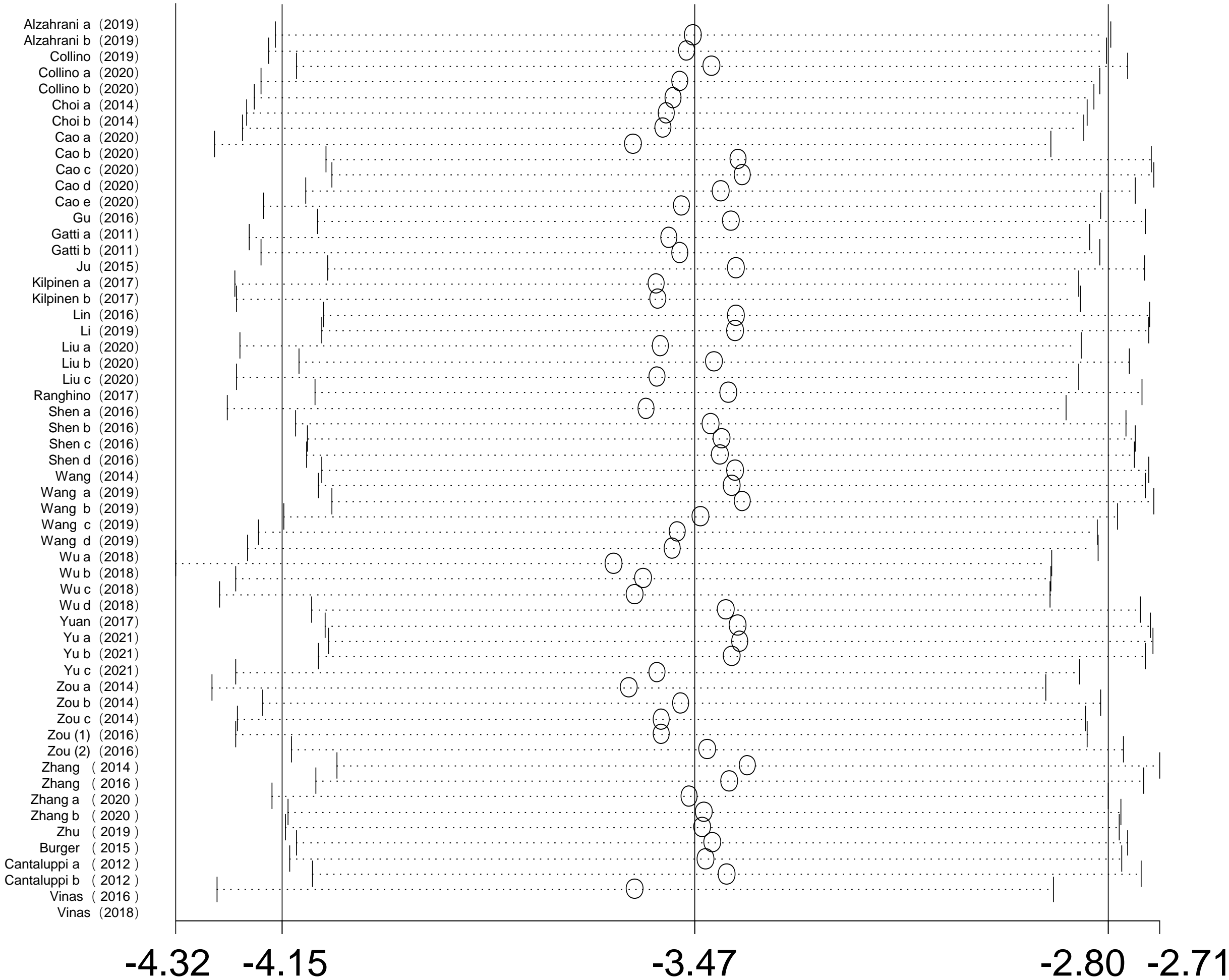

Supplement: Supplementary file 4 — Additional file 4. Sensitivity analysis. [file 13643_2022_2003_MOESM4_ESM.pdf]

Egger's publication bias plot

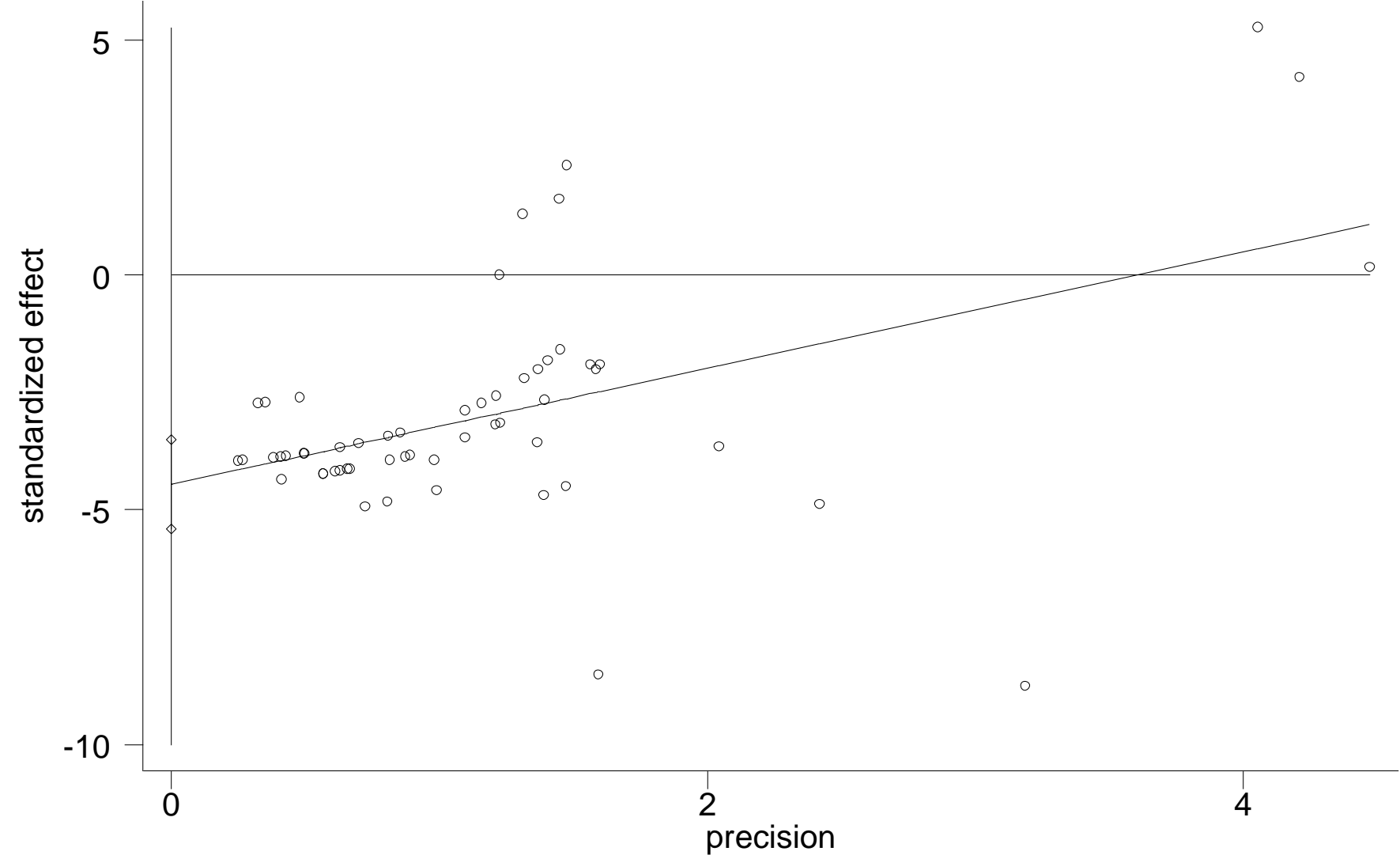

Supplement: Supplementary file 5 — Additional file 5. Eggers test. [file 13643_2022_2003_MOESM5_ESM.pdf]

Filled funnel plot with pseudo 95% confidence limits

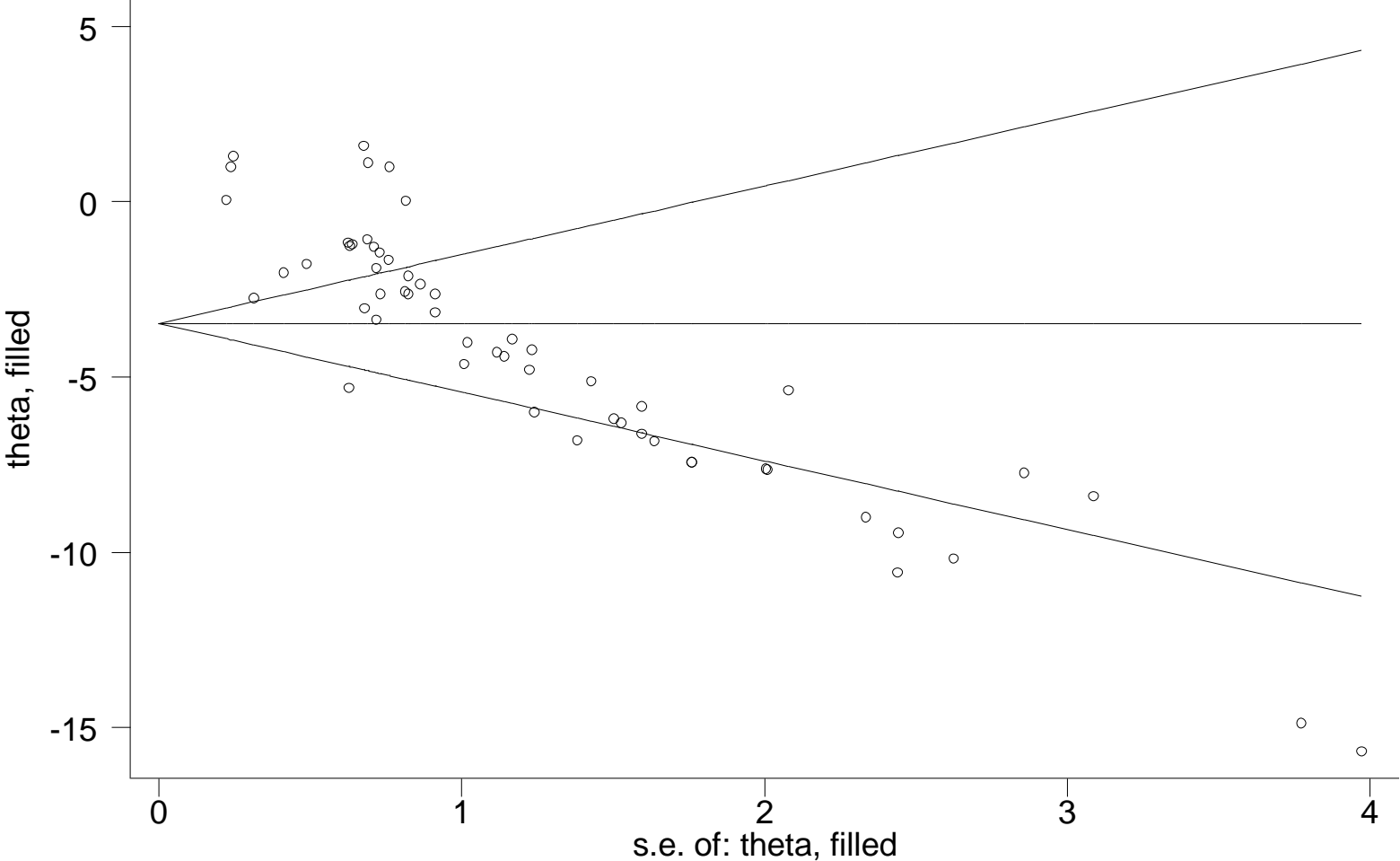

Supplement: Supplementary file 6 — Additional file 6. Trim and fill analysis. [file 13643_2022_2003_MOESM6_ESM.pdf]
